# Reduced learning bias towards the reward context in medication-naive first-episode schizophrenia patients

**DOI:** 10.1186/s12888-021-03682-5

**Published:** 2022-02-16

**Authors:** Xiaoyan Cheng, Lingling Wang, Qinyu Lv, Haisu Wu, Xinxin Huang, Jie Yuan, Xirong Sun, Xudong Zhao, Chao Yan, Zhenghui Yi

**Affiliations:** 1 Shanghai Mental Health Center, Shanghai Jiao Tong University School of Medicine, 600 South Wanping Road, Shanghai, China; 2 Clinical Research Center for Mental Disorders, Shanghai Pudong New Area Mental Health Center, School of Medicine, Tongji University, Shanghai, China; 3 Neuropsychology and Applied Cognitive Neuroscience Laboratory, CAS Key Laboratory of Mental Health, Institute of Psychology, Chinese Academy of Sciences, Beijing, China; 4 Department of Psychology, University of Chinese Academy of Sciences, Beijing, China; 5 Key Laboratory of Brain Functional Genomics (MOE&STCSM), Affiliated Mental Health Center (ECNU), School of Psychology and Cognitive Science, East China Normal University, 3663 North Zhongshan Road, Shanghai, 200062 China

**Keywords:** Reinforcement Learning, Reward context, Prediction error, Expected value, Negative symptom, Medication-naïve

## Abstract

**Background:**

Reinforcement learning has been proposed to contribute to the development of amotivation in individuals with schizophrenia (SZ). Accumulating evidence suggests dysfunctional learning in individuals with SZ in Go/NoGo learning and expected value representation. However, previous findings might have been confounded by the effects of antipsychotic exposure. Moreover, reinforcement learning also rely on the learning context. Few studies have examined the learning performance in reward and loss-avoidance context separately in medication-naïve individuals with first-episode SZ. This study aimed to explore the behaviour profile of reinforcement learning performance in medication-naïve individuals with first-episode SZ, including the contextual performance, the Go/NoGo learning and the expected value representation performance.

**Methods:**

Twenty-nine medication-naïve individuals with first-episode SZ and 40 healthy controls (HCs) who have no significant difference in age and gender, completed the Gain and Loss Avoidance Task, a reinforcement learning task involving stimulus pairs presented in both the reward and loss-avoidance context. We assessed the group difference in accuracy in the reward and loss-avoidance context, the Go/NoGo learning and the expected value representation. The correlations between learning performance and the negative symptom severity were examined.

**Results:**

Individuals with SZ showed significantly lower accuracy when learning under the reward than the loss-avoidance context as compared to HCs. The accuracies under the reward context (90%win- 10%win) in the Acquisition phase was significantly and negatively correlated with the Scale for the Assessment of Negative Symptoms (SANS) avolition scores in individuals with SZ. On the other hand, individuals with SZ showed spared ability of Go/NoGo learning and expected value representation.

**Conclusions:**

Despite our small sample size and relatively modest findings, our results suggest possible reduced learning bias towards reward context among medication-naïve individuals with first-episode SZ. The reward learning performance was correlated with amotivation symptoms. This finding may facilitate our understanding of the underlying mechanism of negative symptoms. Reinforcement learning performance under the reward context may be important to better predict and prevent the development of schizophrenia patients’ negative symptom, especially amotivation.

**Supplementary Information:**

The online version contains supplementary material available at 10.1186/s12888-021-03682-5.

## Background

Amotivation is a core negative symptom of schizophrenia (SZ) [1] and closely correlated with poor clinical and functional outcomes [2–4]. Reinforcement learning (RL) involves assigning values to stimuli for driving motivated behaviours, which is believed to contribute to the underlying mechanisms for amotivation in SZ [5, 6].

Prediction Error (PE) signal and the expected value (EV) representation are essential in the operation of RL [7]. PE signals indicate the difference between the expected reward value and the received reward value [7]. Dopamine (DA) neurons located in the basal ganglia pathway generate PE signals by increasing phasic firing rates if the actual outcome is better than the expected outcome (positive PE), or decreasing phasic firing rates if the actual outcome is worse than the expected outcome (negative PE). After a serial trials, PE signals could enhance (Go learning) or reduce (NoGo learning) the association strength between the stimulus and action [7]. Defective operation of PE signals may result in dysfunctional value assignment. Indeed, patients with SZ have been found to exhibit altered Go learning but preserved NoGo learning [8–11]. Neuroimaging studies have also demonstrated blunted neural responses towards positive PEs in the striatum, the midbrain and other limbic regions [12–14]. Impaired Go learning, coupled with intact NoGo learning, appears to characterize the underpinning of amotivation in SZ [6].

The flexible inner representations of the expected value of the stimuli mainly involve the prefrontal cortex [7]. Clinical patients such as those with SZ have impaired prefrontal functions, and may assign the same EV to all positive PEs regardless of whether PEs are associated with reward or loss-avoidance [15]. In medicated patients with SZ, impaired EV representation has been found at both the behavioural [16–19] and brain functioning levels [20].

Despite the important role of Go/NoGo learning and the EV representation, the context in which RL is initiated is also an important factor in determining RL performance, since the context value sets the “reference point” to which an outcome would be compared, for updating and modifying value assignment [21]. For example, in contexts which entail an overall negative value (i.e., losses), successful trials of loss-avoidance will result in positive PEs. Evidence from behavioural sciences suggests that the different weightings of loss and reward are taken as a hardwired feature of people’s decision making [22]. Various biological mechanisms have been found to underlie reward−loss asymmetry, including genotypes [23], hormonal levels [24] and brain activation during reward processing [25]. Consequently, if one fails to adopt a context-dependent strategy, dysfunctional RL may occur. Indeed, one functional Magnetic Resonance Imaging (fMRI) study found reduced PE responses in unmedicated SZ patients in reward but not loss contexts within various regions including the medial prefrontal cortex, the striatum, and the medial temporal lobe [21].

The above studies have suggested that SZ patients may have impaired RL performance and the impairments may contribute to amotivation symptoms. However, findings on RL in SZ patients have been confounded by effects of medications which are DA-blocking agents. Evidence supports that antipsychotic medications exposure can affect the DA system and thus RL. Eisenegger et al. [26] demonstrated that sulpiride, a D2-like DA antagonist, can disrupt approaching behaviour towards rewards in healthy volunteers, whereas their loss-avoidance behaviour was unaffected [26]. This is also supported by one fMRI study showing that SZ patients receiving higher dosages of antipsychotic medications exhibited lower PE signals in the basal ganglia [27]. Moreover, previous studies on medicated patients with SZ revealed an association between negative symptoms and RL impairment [6, 8, 11, 17, 28], while studies recruiting unmedicated patients with SZ failed to find an association of negative symptoms with RL [29].

To address these limitations, this study examined RL performance in medication-naïve patients with first-episode SZ, using the well-validated paradigm of the Gain and Loss-Avoidance (GLA) task [17]. The GLA task taps into all the above three important aspects of RL. It should be noted that, in the majority of previous studies, the Go/NoGo learning index was conflated with the reward/loss-avoidance context. The Go learning was associated with reward receipt (reward context) and the NoGo learning was associated with loss (loss-avoidance context) [9]. The GLA task enables us to disentangle these two indexes. Given that the previous studies which found impaired Go learning but intact NoGo learning failed to differentiate the effects of reward/loss-avoidance contexts on RL, we hypothesized that SZ patients would could be showing impaired RL in the reward context coupled with intact RL in the loss-avoidance context, but not impaired Go learning coupled with intact NoGo learning. For EV representation, based on previous findings in medicated sample [6, 8, 16, 30], we hypothesized that medication-naïve patients with first-episode SZ would exhibit deficits in representing EV. We also hypothesized that greater RL impairment would be correlated with severe negative symptoms, predominantly in the amotivation dimension in SZ patients.

## Methods

### Participants

Twenty-nine patients with medication-native first-episode SZ diagnosed according to Diagnostic and Statistical Manual of Mental Disorders, Fourth Edition (DSM–IV) [31] criteria were recruited from the Shanghai Mental Health Centre. None of clinical participants were taking any antipsychotic medications. The exclusion criteria were 1) history of other psychiatric disorder; 2) history of any neurological disorders; 3) acute exacerbations of psychotic symptoms; and 4) history of substance abuse in the past 30 days. Forty healthy individuals were recruited as healthy controls (HCs) from the community via distributing fliers and social media platforms. The exclusion criteria for HCs were 1) history of psychosis or neurological disorders; 2) family history of psychotic disorder; and 3) lifetime history of substance abuse. The study was approved by the Ethics Committee of the Shanghai Mental Health Centre (2017-19R). All participants provided written informed consent.

### Gain and loss-avoidance task

**Fig. 1:**
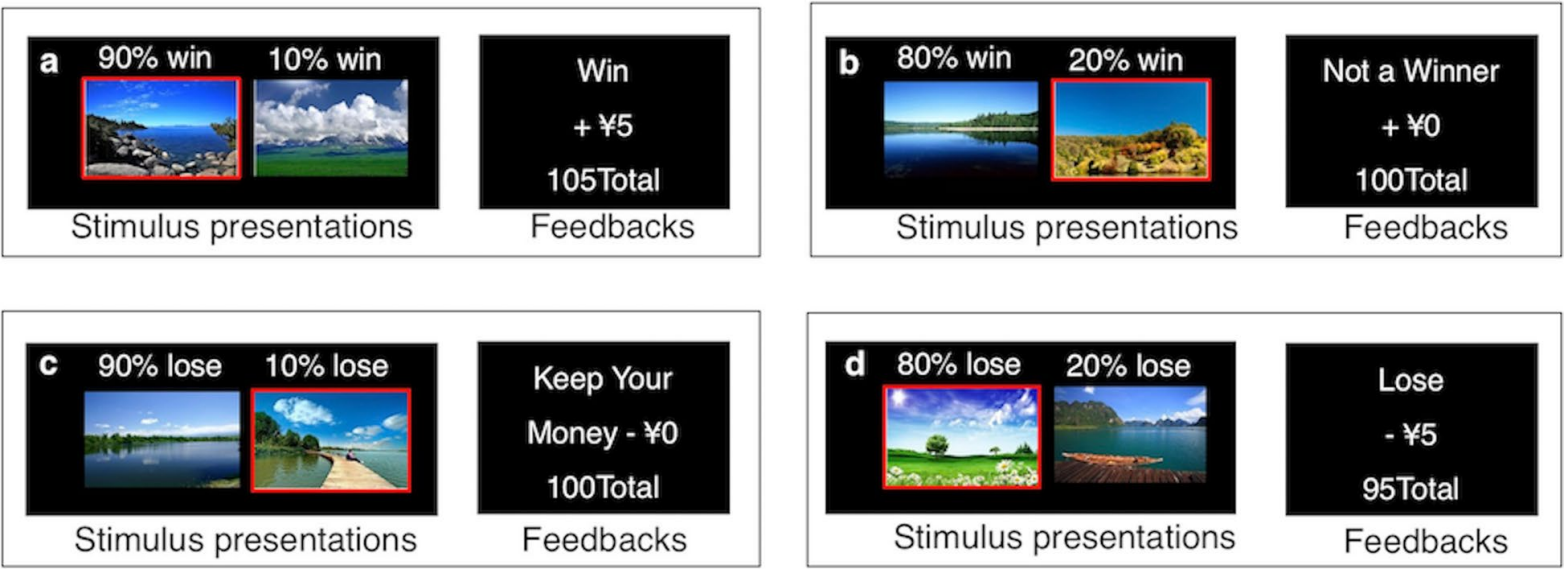
Stimuli and feedbacks in the Acquisition Phase of the GLA task. **a** Feedback delivered after a correct choice (indicated by a red border) in the reward trials. **b** Feedback delivered following an incorrect choice in the reward trials. **c** Feedback delivered following a correct choice in the loss-avoidance trials. **d** Feedback delivered following an incorrect choice in the loss-avoidance trials

The adapted version of the GLA task was developed based on Gold and colleagues’s [17] paradigm. In our GLA paradigm, eight landscape pictures (Figure 1) were used as stimuli.

These pictorial cues were chosen as stimuli (cue) because we tested the arousal and valence ratings they could induce in 16 college students, and the results showed that the 8 pictures were comparable (see Additional file 1 Table S1).

The GLA contained two phases: the acquisition phase and the transfer phase. In the acquisition phase, four different pairs of cues were presented pseudo-randomly. Two pairs were associated with potential rewards, the other two with potential losses. Once presented with a pair of cues, participants were instructed to select the picture that was most likely to either (1) earn money (reward trials) or (2) avoid losing money (loss-avoidance trials). Feedback regarding the outcome was delivered based on the designated reinforcement property of each cue (e.g., Frequent-winner, 90% win: 90% chance of winning ¥5 and 10% of getting ¥0 (Figure 1)). Each pair of cues was presented 10 times in each block. There were four blocks, which resulted in a total of 160 trials. In the transfer phase, the previously learned four pairs of cues and 24 novel pairs of cues were pooled together and presented randomly (see Additional file 2 Table S2). Participants were instructed to select the optimal cue in each pair. In this phase, no feedback would be delivered. Each original pair was presented four times, and each novel pair presented twice. Monetary reward was calculated based on task performance in the Transfer phase, and could range from ¥30 to ¥50 (US$4-7).

### Cognitive and clinical measures

All participants completed the information, arithmetic, similarities and digit span (forward and backward) subtests of the Wechsler Adult Intelligence Scale–Chinese version (WAIS-RC) for estimation of intelligence quotient (IQ). We also administered the Positive and Negative Syndrome Scale (PANSS) [32] and the SANS [33] to patients.

### Statistical analysis

The task performance accuracy was calculated as the percentage of correct responses in choosing an item from the pair which could generate more reward or avoiding more loss in the acquisition and transfer phases. Trials with response time (RT) shorter than 100ms (0.5% trials on average) were deemed invalid and were excluded from the analysis.

Participants’ performance accuracy in the acquisition phase was analyzed using a four-way repeated-measure Analysis of Variance (ANOVA), with Context (reward vs. loss-avoidance), Probability (80% vs. 90%), Block (1 - 4) and Group (SZ vs. HC) as independent variables.

During the transfer phase, two indices (i.e., the Go learning and the NoGo learning) were generated, based on the accuracy of performance across contexts. Pairs consisting of a most frequently reinforced item (i.e., 90% win / 90% loss-avoidance) but not a most infrequently-reinforced item (i.e., 10% win / 10% loss-avoidance) were defined as “Go learning” pairs, which including 90% win vs 80% win, 90% win vs 20% win, 90% loss-avoidance vs 80% loss-avoidance and 90% loss-avoidance vs 20% loss-avoidance; whereas pairs which contained one most infrequently-reinforced item (10% win / 10% loss-avoidance) rather than most frequently reinforced item (i.e., 90% win / 90% loss-avoidance) were defined as “NoGo learning” pairs (i.e., 10% win vs 80% win, 10% win vs 20% win, 10% loss-avoidance vs 80% loss-avoidance and 10% loss-avoidance vs 20% loss-avoidance). Based on the accuracy of the Go and NoGo learning index, we conducted a repeated-measure ANOVA with Go/NoGo (Go vs. NoGo) as within-subject factor and Group as between-subject factor. To further examine the different performance of Go/NoGo learning, the difference scores in Go and NoGo learning accuracy were also extracted and tested for group differences.

For the reward context index, we averaged all the novel pair with both winning cues (i.e., 90% win vs 80% win, 90% win vs 20% win, 80% win vs 10% win and 10% win vs 20% win). Similarly, for the loss-avoidance context, four pairs including 90% loss-avoidance vs 80% loss-avoidance, 90% loss-avoidance vs 20% loss-avoidance, 80% loss-avoidance vs 10% loss-avoidance and 10% loss-avoidance vs 20% loss-avoidance were averaged. A repeated-measure ANOVA was then conducted with Context (Reward vs. Loss-avoidance) as the within-subject factor and Group as the between-subject factor. The difference scores in reward and loss-avoidance context accuracy were also calculated in order to examine the contextual learning bias towards either context. Group differences were tested using independent t tests.

To estimate participants’ ability in representing the EV, we calculated the accuracy of performance in four types of pairs in the transfer phase (i.e., 80% loss-avoidance vs 80% win, 90% loss-avoidance vs 90% win, 20% win vs 20% loss-avoidance and 10% win vs 10% loss-avoidance). Notably, these four pairs contained cues having same valence and probability of PE, but different EVs. Given that participants likely encountered items with high probability more often rather than those with low probability during the Acquisition phase, we generated two separate indices for EV (i.e., high probability EV index and low probability EV index). Moreover, given that the expected value indices are relied on the assumption of equal utilization of the positive/negative PEs in the reward and loss-avoidance context, the difference scores between reward and loss-avoidance context accuracy was taken as a covariate in the univariate ANOVAs to determine the group difference.

Given the gender difference in RL [34] and the close relationship between working memory (WM) and RL [35, 36], participants’ gender and WM performance (backward digit span) were entered as covariates in all the analyses. We took the WM performance so as to ascertain the effect of diagnosis on reinforcement learning without the confounding effect of poor WM associated with SZ patients. However, it is possible that covarying will reduce the ability of detecting diagnosis effects. Thus, we also did an exploratory analysis without the covariates and the results remained significant.

Partial correlations were used to examine the relationship between RL and clinical symptoms in terms of amotivation and anhedonia severity (Scale for the Assessment of Negative Symptoms (SANS) avolition and anhedonia subscale scores) in SZ participants, while controlling for gender and WM (backward digit span). We also examined the relationship between RL indices and WM in clinical participants, while controlling for the gender effect. The False Discovery Rate (FDR) corrections were applied. Greenhouse-geisser correction was used for results that did not meet the sphericity assumption.

## Results

### Demographics, cognitive functions and clinical characteristics

As shown in Table 1, the two groups did not differ in age, gender, education level, IQ estimates and WM performance (*p*s > .05).

**Table 1:** Demographics, cognitive functions and clinical characteristics

Variables^a^	SZ (*n*=29)	HC (*n*=40)	*χ2/t* (df)	*p-value*	Cohen’s d
Age (years)	24.69 (6.16)	25.78 (3.54)	-0.85^b^ (41.32)	0.40	-0.23
Male gender, n (%)	19(65.5)	17(42.5)	3.57^c^ (67)	0.06	0.23^d^
Length of Education (years)	13.59 (2.72)	14.15 (4.12)	-0.68^b^ (66.5)	0.50	-0.17
IQ estimates	107.52 (12.74)	111.55 (15.54)	-1.15^b^ (67)	0.26	0.28
Digit span_forward	8.93 (1.13)	8.77 (1.11)	0.59^b^ (66)	0.56	0.14
Digit span_backward	6.03 (1.57)	6.23 (1.69)	-0.49^b^ (66)	0.63	-0.12
Duration (months)	10.76 (10.08)				
SANS_Flattened Affect	12.38 (9.09)				
SANS_Alogia	5.48 (5.24)				
SANS_avolition	9.48 (3.36)				
SANS_ anhedonia	13.24 (4.95)				
SANS_attention	4.83 (3.10)				
SANS_Total	45.41 (20.58)				
PANSS_negative	23.55 (8.83)				
PANSS_positive	18.76 (7.57)				
PANSS_disorganize	44.34 (10.01)				
PANSS_Total	86.66 (21.10)				

*Note*: *SZ* schizophrenia patients, *HC* healthy control, *IQ* Intelligence Quotient, *SANS* Schedule for the Assessment of Negative Symptoms, *PANSS* Positive and Negative Syndrome Scale

^a^Variables were presented in mean and SD except gender. Gender was presented in number and percentage

^b^Independent sample t test

^c^Chi-square

^d^φ (phi)

### Participants’ performance in the acquisition phase

**Fig. 2:**
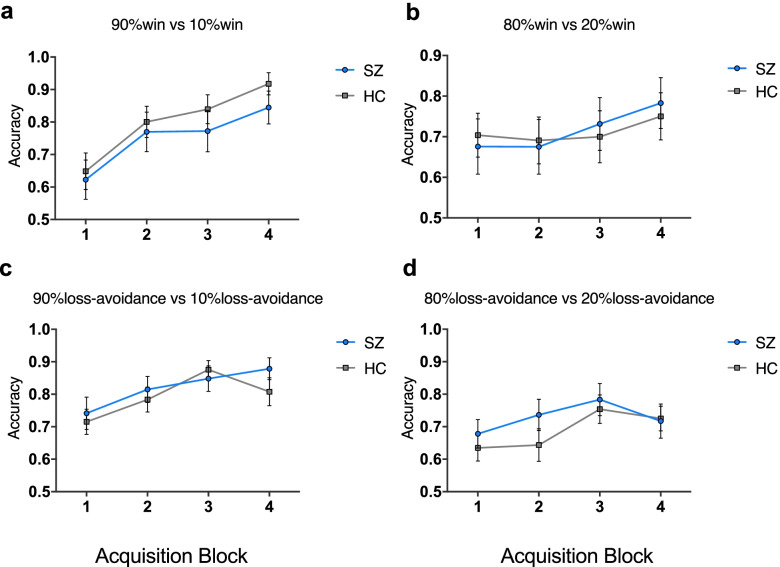
Performance at each block in the Acquisition Phase. **a** Performance at pair of 90% win vs 10% win. **b** Performance at pair of 80% win vs 20% win. **c** Performance at pair of 90% loss-avoidance vs 10% loss-avoidance. **d** Performance at pair of 80% loss-avoidance vs 20% loss-avoidance

The four-way ANOVA revealed a significant main effect of Block (*F*_*2.57,171.83*_ = 19.32, *p* <.001, *η*^*2*^ = 0.22), indicating that participants’ learning accuracy improved steadily over time (Figure 2).

The main effect of Probability was significant (*F*_*1,67*_ =6.16, *p* = .02, *η*^*2*^ = 0.08), suggesting the accuracies improved as the probability increased. Both groups’ performances in Block 4 were significantly better than random level (*ps* <.001). The Group-by-Context interaction failed to reach significance (*F*_*1,67*_ = 1.25, *p* = .27, *η*^*2*^ = 0.02). Main effect of Context (*F*_*1,67*_ =0.26, *p* = .62, *η*^*2*^ = 0.004) and Group (*F*_*1,67*_ <0.001, *p* = .99, *η*^*2*^ < 0.001) also failed to reach statistical significance. The Context-by-Block interaction (*F*_*2.58,173.00*_ = 3.44, *p* = .02, *η*^*2*^ = 0.05) and the Probability-by-Block interaction (*F*_*2.47,165.17*_ = 4.13, *p* = .007, *η*^*2*^ = 0.06) were significant. None of the other 3-way interactions and the 4-way interaction were significant.

### Participants’ performance in the transfer phase

**Fig. 3:**
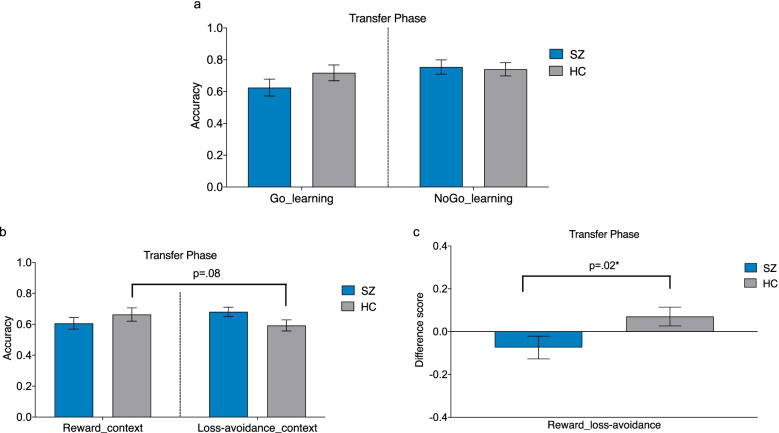
Group difference in the Transfer phase. **a** Accuracies of Go/NoGo learning. **b** Accuracies of reward/loss-avoidance context learning. **c** Difference scores in reward and loss-avoidance context learning accuracy. * *p* < 0.05

When the Go and NoGo learning indices were subjected to repeated measure ANOVA, the main effect of Group was not significant (*F*_1,63_ = 0.46, *p* =.50, *η*^*2*^ = 0.01), suggesting that participants with SZ did not show a general learning impairment relative to controls. The Group-by-Go/NoGo interaction was not significant (*F*_1,63_ = 1.89, *p* =0.17, *η*^*2*^ = 0.03, Figure 3A), showing that both groups have comparable performance in the Go and NoGo learning. Also, no significant group difference was found on the difference scores between Go-NoGo learning accuracy (*F*_1,63_ = 1.89, *p* =.17, *η*^*2*^ = 0.03). However, the difference scores between the reward to loss-avoidance context yield a significant group difference (*F*_1,62_ = 5.60, *p* =.02, *η*^*2*^ = 0.08, Figure 3C). Participants with SZ showed significantly reduced learning bias from reward context than HCs. The Group-by-Context interaction was also found significant (*F*_1,62_ = 5.60, *p* =.02, *η*^*2*^ = 0.08, see Figure 3B). Further analysis indicated HCs, but not SZs, performed better in the reward context than loss-avoidance context. The main effect of Context (*F*_1,62_ = 0.19, *p* =.66, *η*^*2*^ = 0.003) and Group (*F*_1,62_ = 0.05, *p* =.83, *η*^*2*^ = 0.001) were not significant.

When the EV indices were subjected to ANOVAs, SZ participants and HCs showed comparable EV indices both in the low (*F*_1,64_ = 0.001, *p=.97, η*^*2*^ < 0.001) and high probability conditions (*F*_1,64_ = 1.66, *p=.20, η*^*2*^ = 0.03).

### Correlations between RL performance and clinical measures in medication-naïve participants with first-episode SZ

A significant and negative correlation was found between the accuracy of learning in reward context (10% win - 90% win) across the acquisition phase and the avolition subscale score of the SANS (*r*_*23*_ =-0.54, *p*_*FDR-corrected*_ =.004).

## Discussion

The present study investigated the multiple aspects of RL in medication-naïve patients with first-episode SZ. Despite the limited sample size and modest findings, we found preliminary evidence of SZ patients showing reduced contextual bias towards the reward context. Furthermore, the reward context learning performance was correlated with avolition symptoms of individuals with SZ. We found no evidence for dysfunction in Go/NoGo learning and EV representations in patients with SZ.

In medication-naïve SZ patients, the results showed intact Go and NoGo learning relative to controls. Similarly, recent studies also found intact positive and negative PE-driven learning in patients with chronic SZ [37]. On the other hand, a few previous studies on chronic SZ reported that both the Go and NoGo learning were impaired [38, 39]. However, the proposed selectively impaired Go but intact NoGo learning was not consistently found in patients with SZ. Compared with previous evidence, our findings were unlikely to be confounded by medication effect on the DA systems, and suggested that the Go and NoGo learning in SZ patients were largely intact. The role of Go and NoGo learning in SZ could vary among different stages of schizophrenia.

Our findings indicated a possible deficit in reduced learning bias towards the reward context in patients with SZ, which is consistent with previous study found more pronounced impairment in reward context among unmedicated SZ patients [40]. Indeed, previous fMRI results suggested that attenuated PE response in unmedicated patients with SZ in the medial prefrontal cortex under the reward but not loss-avoidance context [21, 41]. It also dovetails with studies using the same GLA task on medicated patients with chronic SZ, which were found to have poorer performance in reward than loss-avoidance trials [17, 42, 43]. The attenuated learning from rewards than loss-avoidance context in medication-naïve SZ patients, together with similar findings observed in chronic medicated SZ patients, may suggest a persistent dysregulation throughout the course of illness. Our findings of correlation analysis also suggest a positive relationship between the learning performance under the reward context and avolition symptom. This finding may indicate that reduced learning bias toward reward may related to the more severe amotivation symptoms.

Regarding EV representation, we found no evidence for impaired EV representation in medication-naïve first-episode SZ patients, consistent with earlier results using medicated SZ samples [28]. Our participants with SZ showed comparable preferences for reward stimuli over loss-avoidance stimuli as controls. Similar findings have been reported in individuals with ultra-high risk for SZ of their intact prefrontal activity during PE signaling and reward anticipation [44–46], suggesting that people at the very early stage of the SZ spectrum are capable of representing EV. EV performance and prefrontal activation while evaluating reward outcomes have repeatedly been found to be correlated with the severity of negative symptoms [29]. However, in our study, as negative symptoms were unlikely to attributable to medication effects, this relationship was not found. A similar study on brain activity in medication-naïve SZ patients during reward anticipation also did not find any significant correlation between prefrontal activation and negative symptoms [29]. According to the theory proposed by Waltz and Gold [15], although both unmedicated and medicated SZ patients have disrupted RL, the aberrant learning observed in medicated chronic SZ may more likely be due to faulty EV representation rather than dysfunctional PE utilization, while the latter mechanism may be more applicable to unmedicated SZ patients. Such an account posits that EV is strongly linked to negative symptoms in chronic SZ, leading to persistence of these symptoms throughout the illness. Although our sample size was relatively small, our preliminary results suggested that EV may play an important role in maintaining the negative symptoms rather than causing them.

This study has several limitations. First, the sample size was relatively small many of our results were modest in magnitudes, which might have limited statistical power. Moreover, given the limited sample size, it is possible that the current sample may not cover the full populations within the medication-naïve first-episode schizophrenia patients. Our sample was biased by a certain degree of highly-educated and young patients and we encouraged the readers to interpret the results with cautious. Future studies with larger sample size and a more representative sample are in great need to verify and replicate the present results. Second, our paradigm was limited by having a small number of trials. Future studies are required to verify the results with more trials. The GLA task was monetary in nature. Future developments of experimental paradigms imbedded in the social and interpersonal context may further promote the investigation of patients’ learning from social reward and social pleasure. Third, our sample of medication-naïve first-episode SZ patients apparently had a low level of anhedonia and amotivation. In order to understand the role of reinforcement learning in the formation of amotivation, future studies should recruit first-episode SZ patients with prominent negative symptoms.

## Conclusions

In conclusion, we found preliminary evidence of a lack of learning bias towards the reward context in medication-naïve first-episode SZ patients. Performance under the reward context was negatively correlated with avolition symptoms measured by the SANS. In addition, we find patients with SZ demonstrated preserved EV representation and Go/NoGo learning in the early stages of the disease. Impaired reinforcement learning under the reward context in this very early case of SZ may indicate that it could serve as a viable starting point to better predict and prevent the developments of patients’ negative symptoms.

## Supplementary Information

**Additional file 1: Table S1.** Valence and Arousal of GLA Task Stimuli.

**Additional file 2: Table S2.** Pairs in the Gain vs Loss-Avoidance (GLA) task.

## Data Availability

The datasets used and/or analysed during the current study are available from the corresponding author on reasonable request.

## Abbreviations

ANOVA: Analysis of Variance
DA: Dopamine
DSM–IV: Diagnostic and Statistical Manual of Mental Disorders, Fourth Edition
EV: Expected Value
FDR: False Discovery Rate
fMRI: Functional Magnetic Resonance Imaging
GLA: Gain and Loss-Avoidance
HCs: Healthy Controls
IQ: Intelligence Quotient
PANSS: Positive and Negative Syndrome Scale
PE: Prediction Error
RL: Reinforcement Learning
RT: Response Time
SANS: Scale for the Assessment of Negative Symptoms
SZ: Schizophrenia
WAIS-RC: Wechsler Adult Intelligence Scale–Chinese version
WM: Working Memory

## References

[1] Strauss GP, Bartolomeo LA, Harvey PD (2021). Avolition as the core negative symptom in schizophrenia: relevance to pharmacological treatment development. NPJ Schizophr.

[2] Strauss GP, Horan WP, Kirkpatrick B, Fischer BA, Keller WR, Miski P (2013). Deconstructing negative symptoms of schizophrenia: avolition–apathy and diminished expression clusters predict clinical presentation and functional outcome. J Psychiatr Res.

[3] Chang WC, Ho RWH, Tang JYM, Wong CSM, Hui CLM, Chan SKW (2019). Early-stage negative symptom trajectories and relationships with 13-year outcomes in first-episode nonaffective psychosis. Schizophr Bull.

[4] Najas-Garcia A, Gómez-Benito J, Huedo-Medina TB (2018). The relationship of motivation and neurocognition with functionality in schizophrenia: a meta-analytic review. Community Ment Health J.

[5] Gold JM, Waltz JA, Prentice KJ, Morris SE, Heerey EA (2008). Reward processing in schizophrenia: a deficit in the representation of value. Schizophr Bull.

[6] Strauss GP, Waltz JA, Gold JM (2014). A review of reward processing and motivational impairment in schizophrenia. Schizophr Bull.

[7] Frank MJ, Claus ED (2006). Anatomy of a decision: striato-orbitofrontal interactions in reinforcement learning, decision making, and reversal. Psychol Rev.

[8] Strauss GP, Frank MJ, Waltz JA, Kasanova Z, Herbener ES, Gold JM (2011). Deficits in positive reinforcement learning and uncertainty-driven exploration are associated with distinct aspects of negative symptoms in schizophrenia. Biol Psychiatry.

[9] Waltz JA, Frank MJ, Robinson BM, Gold JM (2007). Selective reinforcement learning deficits in schizophrenia support predictions from computational models of striatal-cortical dysfunction. Biol Psychiatry.

[10] Waltz JA, Frank MJ, Wiecki TV, Gold JM (2011). Altered probabilistic learning and response biases in schizophrenia: behavioral evidence and neurocomputational modeling. Neuropsychology..

[11] Yılmaz A, Simsek F, Gonul AS (2012). Reduced reward-related probability learning in schizophrenia patients. Neuropsychiatr Dis Treat.

[12] Gradin VB, Kumar P, Waiter G, Ahearn T, Stickle C, Milders M (2011). Expected value and prediction error abnormalities in depression and schizophrenia. Brain..

[13] Murray GK, Corlett PR, Clark L, Pessiglione M, Blackwell AD, Honey G (2008). Substantia nigra/ventral tegmental reward prediction error disruption in psychosis. Mol Psychiatry.

[14] Waltz JA, Schweitzer JB, Gold JM, Kurup PK, Ross TJ, Jo Salmeron B (2009). Patients with schizophrenia have a reduced neural response to both unpredictable and predictable primary reinforcers. Neuropsychopharmacol..

[15] Waltz JA, Gold JM, Simpson EH, Balsam PD (2016). Motivational deficits in schizophrenia and the representation of expected value. Behavioral neuroscience of motivation.

[16] Barch DM, Treadway MT, Schoen N (2014). Effort, anhedonia, and function in schizophrenia: reduced effort allocation predicts amotivation and functional impairment. J Abnorm Psychol.

[17] Gold JM, Waltz JA, Matveeva TM, Kasanova Z, Strauss GP, Herbener ES (2012). Negative symptoms and the failure to represent the expected reward value of actions: behavioral and computational modeling evidence. Arch Gen Psychiatry.

[18] Brown EC, Hack SM, Gold JM, Carpenter WT, Fischer BA, Prentice KP (2015). Integrating frequency and magnitude information in decision-making in schizophrenia: An account of patient performance on the Iowa Gambling Task. J Psychiatr Res.

[19] Hernaus D, Gold JM, Waltz JA, Frank MJ (2018). Impaired expected value computations coupled with overreliance on stimulus-response learning in schizophrenia. Biol Psychiatr Cogn Neurosci Neuroimag.

[20] Waltz JA, Xu Z, Brown EC, Ruiz RR, Frank MJ, Gold JM (2018). Motivational deficits in schizophrenia are associated with reduced differentiation between gain and loss-avoidance feedback in the striatum. Biol Psychiatr Cogn Neurosci Neuroimag.

[21] Reinen JM, Van Snellenberg JX, Horga G, Abi-Dargham A, Daw ND, Shohamy D (2016). Motivational context modulates prediction error response in schizophrenia. Schizophr Bull.

[22] Koszegi B, Rabin M (2006). A model of reference-dependent preferences. Q J Econ.

[23] Frydman C, Camerer C, Bossaerts P, Rangel A (2011). MAOA-L carriers are better at making optimal financial decisions under risk. Proc R Soc B.

[24] Chumbley JR, Krajbich I, Engelmann JB, Russell E, Van Uum S, Koren G (2014). Endogenous cortisol predicts decreased loss aversion in young men. Psychol Sci.

[25] Sokol-Hessner P, Camerer CF, Phelps EA (2013). Emotion regulation reduces loss aversion and decreases amygdala responses to losses. Soc Cogn Affect Neurosci.

[26] Eisenegger C, Naef M, Linssen A, Clark L, Gandamaneni PK, Müller U (2014). Role of dopamine D2 receptors in human reinforcement learning. Neuropsychopharmacol..

[27] Insel C, Reinen J, Weber J, Wager TD, Jarskog LF, Shohamy D (2014). Antipsychotic dose modulates behavioral and neural responses to feedback during reinforcement learning in schizophrenia. Cogn Affect Behav Neurosci.

[28] Chang WC, Waltz JA, Gold JM, Chan TCW, Chen EYH (2016). Mild reinforcement learning deficits in patients with first-episode psychosis. Schizophr Bull.

[29] Nielsen MØ, Rostrup E, Wulff S, Bak N, Lublin H, Kapur S (2012). Alterations of the brain reward system in antipsychotic naïve schizophrenia patients. Biol Psychiatry.

[30] Brown JK, Waltz JA, Strauss GP, McMahon RP, Frank MJ, Gold JM (2013). Hypothetical decision making in schizophrenia: the role of expected value computation and “irrational” biases. Psychiatry Res.

[31] Association AP. Diagnostic and statistical manual of mental disorders. 4th ed: American Psychiatric Association; 1994.

[32] Kay SR, Fiszbein A, Opler LA (1987). The positive and negative syndrome scale (PANSS) for schizophrenia. Schizophr Bull.

[33] Andreasen NC (1989). The scale for the assessment of negative symptoms (SANS): conceptual and theoretical foundations. Br J Psychiatry Suppl.

[34] Byrne KA, Worthy DA (2015). Gender differences in reward sensitivity and information processing during decision-making. J Risk Uncertain.

[35] Collins AGE, Brown JK, Gold JM, Waltz JA, Frank MJ (2014). Working memory contributions to reinforcement learning impairments in schizophrenia. J Neurosci.

[36] Collins AGE, Frank MJ (2012). How much of reinforcement learning is working memory, not reinforcement learning? A behavioral, computational, and neurogenetic analysis: working memory in reinforcement learning. Eur J Neurosci.

[37] Albrecht MA, Waltz JA, Frank MJ, Gold JM (2016). Probability and magnitude evaluation in schizophrenia. Schizophr Res Cognition.

[38] Cicero DC, Martin EA, Becker TM, Kerns JG (2014). Reinforcement learning deficits in people with schizophrenia persist after extended trials. Psychiatry Res.

[39] Fervaha G, Agid O, Foussias G, Remington G (2013). Impairments in both reward and punishment guided reinforcement learning in schizophrenia. Schizophr Res.

[40] Moran EK, Gold JM, Carter CS, MacDonald AW, Ragland JD, Silverstein SM, et al. Both unmedicated and medicated individuals with schizophrenia show impairments across a wide array of cognitive and reinforcement learning tasks. Psychological Medicine. Cambridge University Press; 2020;:1–11. 10.1017/S003329172000286X. 10.1017/S003329172000286X PMC8095353 32799938

[41] Schlagenhauf F, Sterzer P, Schmack K, Ballmaier M, Rapp M, Wrase J (2009). Reward feedback alterations in unmedicated schizophrenia patients: relevance for delusions. Biol Psychiatry.

[42] Hartmann-Riemer MN, Aschenbrenner S, Bossert M, Westermann C, Seifritz E, Tobler PN (2017). Deficits in reinforcement learning but no link to apathy in patients with schizophrenia. Sci Rep.

[43] Barch DM, Carter CS, Gold JM, Johnson SL, Kring AM, MacDonald AW (2017). Explicit and implicit reinforcement learning across the psychosis spectrum. J Abnorm Psychol.

[44] Ermakova AO, Knolle F, Justicia A, Bullmore ET, Jones PB, Robbins TW (2018). Abnormal reward prediction-error signalling in antipsychotic naive individuals with first-episode psychosis or clinical risk for psychosis. Neuropsychopharmacol..

[45] Juckel G, Friedel E, Koslowski M, Witthaus H, Özgürdal S, Gudlowski Y (2012). Ventral striatal activation during reward processing in subjects with ultra-high risk for schizophrenia. Neuropsychobiology..

[46] Wotruba D, Heekeren K, Michels L, Buechler R, Simon JJ, Theodoridou A, et al. Symptom dimensions are associated with reward processing in unmedicated persons at risk for psychosis. Front Behav Neurosci. 2014;8:382. 10.3389/fnbeh.2014.00382. 10.3389/fnbeh.2014.00382 PMC4235359 25477792

