# Supplementary material for: Reduced learning bias towards the reward context in medication-naive first-episode schizophrenia patients
Source: BMC Psychiatry. 2022 Feb 16;22:123. doi: 10.1186/s12888-021-03682-5 (PMC8851841; doi:10.1186/s12888-021-03682-5)
Supplement: Supplementary file 1 — Additional file 1: Table S1. Valence and Arousal of GLA Task Stimuli. [file 12888_2021_3682_MOESM1_ESM.doc]

**Additional file 1. Stimulus selection of Gain vs Loss-Avoidance（GLA） Task**

Eight landscape pictures were used as stimuli. We recruited 16 participants (14 major in psychology, 1 in medical science, and 1 in marine engineering) to evaluate the emotional valence and arousal of 16 images by questionnaire. Five images were excluded since they were 1.5 standard deviations away from the average valence and arousal. Researchers then selected 8 pictures that were easy to distinguish from each other among these 11 pictures.

**Table S1. Valence and Arousal of GLA Task Stimuli**

|  | Valence | SD(Valence) | Arousal | SD(Arousal) | Standardized-valence | Standardized-arousal |
| --- | --- | --- | --- | --- | --- | --- |
| Picture1 | 5.40 | .29 | 4.27 | .43 | 0.34 | 1.50 |
| Picture2 | 5.33 | .27 | 3.80 | .45 | 0.09 | 0.54 |
| Picture3 | 5.13 | .24 | 3.73 | .41 | 0.64 | 0.40 |
| Picture4 | 5.40 | .29 | 3.73 | .41 | 0.34 | 0.40 |
| Picture5 | 5.00 | .28 | 3.27 | .43 | 1.13 | 0.56 |
| Picture6 | 5.33 | .27 | 3.20 | .42 | 0.09 | 0.70 |
| Picture7 | 5.27 | .25 | 3.07 | .40 | 0.15 | 0.97 |
| Picture8 | 5.60 | .25 | 3.00 | .52 | 1.07 | 1.11 |
