# Supplementary material for: Reduced learning bias towards the reward context in medication-naive first-episode schizophrenia patients
Source: BMC Psychiatry. 2022 Feb 16;22:123. doi: 10.1186/s12888-021-03682-5 (PMC8851841; doi:10.1186/s12888-021-03682-5)
Supplement: Supplementary file 2 — Additional file 2: Table S2. Pairs in the Gain vs Loss-Avoidance (GLA) task. [file 12888_2021_3682_MOESM2_ESM.doc]

**Additional file 2. Pairs in the GLA task**

Table S2. Pairs in the Gain vs Loss-Avoidance (GLA) task

|  | Pair |
| --- | --- |
| **Acquisition phase** | 90%win vs 10%win |
|  | 80%win vs 20%win |
|  | 90%loss-avoidance vs 10%loss-avoidance |
|  | 80%loss-avoidance vs 20%loss-avoidance |
| **Transfer phase** |  |
| Expected value_high_probability | 80%loss-avoidance vs 80%win |
|  | 90%loss-avoidance vs 90%win |
| Expected value_low_probability | 20%win vs 20%loss-avoidance |
|  | 10%win vs 10%loss-avoidance |
| Reward context | 90%win vs 20%win |
|  | 90%win vs 80%win |
|  | 10%win vs 20%win |
|  | 10%win vs 80%win |
| Loss_avoidance context | 90%loss-avoidance vs 20%loss-avoidance |
|  | 90%loss-avoidance vs 80%loss-avoidance |
|  | 10%loss-avoidance vs 20%loss-avoidance |
|  | 10%loss-avoidance vs 80%loss-avoidance |
| Go learning | 90%win vs 20%win |
|  | 90%win vs 80%win |
|  | 90%loss-avoidance vs 20%loss-avoidance |
|  | 90%loss-avoidance vs 80%loss-avoidance |
| NoGo learning | 10%win vs 20%win |
|  | 10%win vs 80%win |
|  | 10%loss-avoidance vs 20%loss-avoidance |
|  | 10%loss-avoidance vs 80%loss-avoidance |
| Novel pairs | 90%win vs 80%loss-avoidance |
|  | 90%win vs 20%loss-avoidance |
|  | 80%win vs 90%loss-avoidance |
|  | 80%win vs 10%loss-avoidance |
|  | 20%win vs 90%loss-avoidance |
|  | 10%win vs 80%loss-avoidance |
|  | 10%win vs 20%loss-avoidance |
|  | 20%win vs 10%loss-avoidance |
| Old pairs | 80%win vs 20%win  90%win vs 10%win |
|  | 80%loss-avoidance vs 20%loss-avoidance  90%loss-avoidance vs 10%loss-avoidance |
